# Supplementary material for: Modeling glioblastoma heterogeneity as a dynamic network of cell states
Source: Mol Syst Biol. 2021 Sep 16;17(9):e10105. doi: 10.15252/msb.202010105 (PMC8444284; doi:10.15252/msb.202010105)
Supplement: Supplementary file 6 — Source Data for Figure 5 [file MSB-17-e10105-s004.zip › Figure5A_sourcedata/GSEA_3017/nosigdb_stateC.GseaPreranked.1622795002785/FELDMAN_2020_G1.html]

Details for gene set FELDMAN\_2020\_G1[GSEA]

|  || Dataset | state13017 |
| Phenotype | NoPhenotypeAvailable |
| Upregulated in class | na\_pos |
| GeneSet | FELDMAN\_2020\_G1 |
| Enrichment Score (ES) | 0.5685668 |
| Normalized Enrichment Score (NES) | 3.956146 |
| Nominal p-value | 0.0 |
| FDR q-value | 0.0 |
| FWER p-Value | 0.0 |
Table: GSEA Results Summary

  

Fig 1: Enrichment plot: FELDMAN\_2020\_G1      
 Profile of the Running ES Score & Positions of GeneSet Members on the Rank Ordered List

  

| PROBE | GENE SYMBOL | GENE\_TITLE | RANK IN GENE LIST | RANK METRIC SCORE | RUNNING ES | CORE ENRICHMENT || 1 | ATAD2 |  |  | 7 | 0.649 | 0.0164 | Yes |
| 2 | HIST1H4C |  |  | 9 | 0.603 | 0.0417 | Yes |
| 3 | CENPU |  |  | 10 | 0.588 | 0.0683 | Yes |
| 4 | HIST1H1C |  |  | 11 | 0.583 | 0.0946 | Yes |
| 5 | CLSPN |  |  | 17 | 0.561 | 0.1107 | Yes |
| 6 | RRM2 |  |  | 19 | 0.542 | 0.1333 | Yes |
| 7 | HMGB2 |  |  | 20 | 0.534 | 0.1574 | Yes |
| 8 | UBE2T |  |  | 31 | 0.475 | 0.1604 | Yes |
| 9 | HIST1H1B |  |  | 35 | 0.460 | 0.1757 | Yes |
| 10 | GMNN |  |  | 38 | 0.453 | 0.1925 | Yes |
| 11 | ORC6 |  |  | 39 | 0.450 | 0.2127 | Yes |
| 12 | ESCO2 |  |  | 43 | 0.440 | 0.2271 | Yes |
| 13 | PCNA |  |  | 48 | 0.435 | 0.2394 | Yes |
| 14 | TMPO |  |  | 49 | 0.432 | 0.2589 | Yes |
| 15 | HIST1H1E |  |  | 51 | 0.430 | 0.2764 | Yes |
| 16 | TOP2A |  |  | 52 | 0.426 | 0.2957 | Yes |
| 17 | SPC25 |  |  | 53 | 0.415 | 0.3144 | Yes |
| 18 | DHFR |  |  | 55 | 0.405 | 0.3308 | Yes |
| 19 | TYMS |  |  | 60 | 0.390 | 0.3411 | Yes |
| 20 | RRM1 |  |  | 62 | 0.386 | 0.3566 | Yes |
| 21 | SMC2 |  |  | 63 | 0.386 | 0.3740 | Yes |
| 22 | PSIP1 |  |  | 73 | 0.374 | 0.3743 | Yes |
| 23 | DEK |  |  | 74 | 0.373 | 0.3912 | Yes |
| 24 | CENPH |  |  | 77 | 0.366 | 0.4040 | Yes |
| 25 | SMC4 |  |  | 78 | 0.365 | 0.4205 | Yes |
| 26 | GINS2 |  |  | 83 | 0.360 | 0.4294 | Yes |
| 27 | STMN1 |  |  | 85 | 0.357 | 0.4437 | Yes |
| 28 | DUT |  |  | 87 | 0.356 | 0.4579 | Yes |
| 29 | MCM7 |  |  | 91 | 0.352 | 0.4683 | Yes |
| 30 | USP1 |  |  | 92 | 0.352 | 0.4841 | Yes |
| 31 | FEN1 |  |  | 98 | 0.342 | 0.4904 | Yes |
| 32 | NUSAP1 |  |  | 100 | 0.340 | 0.5038 | Yes |
| 33 | H2AFX |  |  | 115 | 0.322 | 0.4926 | Yes |
| 34 | CCDC34 |  |  | 120 | 0.318 | 0.4996 | Yes |
| 35 | HAT1 |  |  | 121 | 0.318 | 0.5139 | Yes |
| 36 | HMGN2 |  |  | 126 | 0.312 | 0.5206 | Yes |
| 37 | DNMT1 |  |  | 127 | 0.311 | 0.5347 | Yes |
| 38 | FBXO5 |  |  | 130 | 0.308 | 0.5449 | Yes |
| 39 | NCAPG |  |  | 137 | 0.301 | 0.5474 | Yes |
| 40 | CENPK |  |  | 139 | 0.299 | 0.5590 | Yes |
| 41 | LMNB1 |  |  | 142 | 0.293 | 0.5686 | Yes |
| 42 | SIVA1 |  |  | 151 | 0.286 | 0.5668 | No |
| 43 | VRK1 |  |  | 169 | 0.275 | 0.5478 | No |
| 44 | MAD2L1 |  |  | 174 | 0.270 | 0.5527 | No |
| 45 | CDK1 |  |  | 191 | 0.255 | 0.5347 | No |
| 46 | TPST2 |  |  | 226 | -0.259 | 0.4838 | No |
| 47 | IGFBP5 |  |  | 384 | -0.311 | 0.2087 | No |
| 48 | PLP1 |  |  | 386 | -0.312 | 0.2209 | No |
| 49 | SCRG1 |  |  | 508 | -0.434 | 0.0177 | No |
| 50 | PTN |  |  | 517 | -0.446 | 0.0231 | No |
| 51 | IGFBP3 |  |  | 538 | -0.493 | 0.0085 | No |
| 52 | S100B |  |  | 561 | -0.561 | -0.0067 | No |
| 53 | CYR61 |  |  | 584 | -0.714 | -0.0150 | No |
| 54 | CTGF |  |  | 588 | -0.782 | 0.0147 | No |
Table: GSEA details [plain text format]

  

Fig 2: FELDMAN\_2020\_G1: Random ES distribution      
 Gene set null distribution of ES for **FELDMAN\_2020\_G1**
